# Half a Century of Controversy: The Neutralist/Selectionist Debate in Molecular Evolution

**DOI:** 10.1093/gbe/evae003

**Published:** 2024-02-05

**Authors:** Nicolas Galtier

**Affiliations:** ISEM, CNRS, IRD, Université de Montpellier, Montpellier, France

**Keywords:** neutral theory, nearly neutral theory, within-species polymorphism, substitution rate, genome size, GC-biased gene conversion

## Abstract

The neutral and nearly neutral theories, introduced more than 50 yr ago, have raised and still raise passionate discussion regarding the forces governing molecular evolution and their relative importance. The debate, initially focused on the amount of within-species polymorphism and constancy of the substitution rate, has spread, matured, and now underlies a wide range of topics and questions. The neutralist/selectionist controversy has structured the field and influences the way molecular evolutionary scientists conceive their research.

**Significance**

Whether genome evolution is mainly driven by natural selection or largely reflects random, nonadaptive processes is arguably the central issue of molecular evolution as a field. Over the last 40 yr, the question has been hotly debated in the 2 Society for Molecular Biology and Evolution journals, *Molecular Biology and Evolution* and *Genome Biology and Evolution*. Initially focusing on the amount of within-species polymorphism and the rate of amino acid substitution, the controversy now touches every aspect of molecular biology, including genome size, content, and function.

## Introduction

This perspective is part of a series of articles celebrating 40 years since our sister journal, *Molecular Biology and Evolution*, was founded (Russo et al. 2024). The perspective is accompanied by virtual issues, a selection of papers on the neutralist/selectionist debate published by *Genome Biology and Evolution* and *Molecular Biology and Evolution*.

The neutral and nearly neutral theories have polarized the field of molecular evolution since they were first proposed by Kimura (1968) and Ohta (1973). The controversy was intense at the end of the 20th century and remains palpable since the beginning of the 21st century. The 2 Society for Molecular Biology and Evolution (SMBE) journals were key vehicles for the expression of these contrasting viewpoints. The neutral theory was based mainly on 2 observations: (i) that within-species genetic polymorphism is substantial and (ii) that proteins evolve at a roughly constant rate. This led Kimura and others (e.g. King and Jukes 1969) to postulate that most of the observable variations at a molecular level were neutral and governed primarily by drift. This view was revolutionary and challenged the dominant idea stating that, because genes determine phenotypes, genes must evolve like phenotypes, i.e. be mainly directed by natural selection. Interestingly, the 2 cornerstones of the neutral theory are still very much alive and important aspects of current molecular evolutionary thinking.

## Within-Species Polymorphism and Lewontin's Paradox

The among-species variation in genetic polymorphism has been the subject of in-depth analyses, mostly revolving around the meaning and pertinence of the concept of effective population size (*N_e_*). The neutral model predicts that within-species polymorphism should equal the product of mutation rate and *N_e_* (Kimura 1971). However, it was quickly realized that measured amounts of polymorphism do not scale proportionally with species census population size, *N_c_*—the so-called Lewontin's paradox (Lewontin 1974; Leffler et al. 2012; Romiguier et al. 2014; Corbett-Detig et al. 2015; Buffalo 2021). Potential explanations for this conundrum include the suggestions that *N_c_* might strongly differ from *N_e_* (Filatov 2019), that *N_e_* might be inversely related to the mutation rate (Lynch 2007), and that recurrent population bottlenecks might result in frequent drops of diversity in otherwise large *N_c_* populations, which never reach equilibrium (Charlesworth and Jensen 2022). These hypotheses account for the narrow range of the level of genetic polymorphism observed across species, while being compatible with the neutral theory.

Another family of explanations for Lewontin's paradox invokes selective effects and, more specifically, linked selection. It is common knowledge that selection applied to a specific locus will also affect genetic variation at neighboring, linked loci. This idea was already present in the early 70s, when the neutral theory was intensely debated. At that time, the literature focused on the suggestion that elevated amounts of polymorphism could be maintained by associative overdominance, i.e. the effect on linked loci of weakly deleterious alleles maintained by mutation pressure (Ohta 1971) or by heterozygote advantage (Kimura 1971). Since then, the focus has moved to the effect on linked loci of strong directional selection, either positive (selective sweeps, Maynard Smith and Haigh 1974; Wiehe and Stephan 1993; Enard et al. 2014) or negative (background selection, Charlesworth et al. 1993). There is convincing evidence that linked directional selection tends to decrease the neutral genetic diversity, particularly in large *N_e_* species (Corbett-Detig et al. 2015; Castellano et al. 2018; Chen et al. 2020). One such piece of evidence is the report in several species of an across-loci correlation between recombination rate and heterozygosity (Begun and Aquadro 1992; Nachman 2001; Elyashiv et al. 2016)—although the most recent literature also points to alternative explanations (Smith et al. 2018; Barroso and Dutheil 2023). When recombination is low or absent, large chunks of DNA cosegregate with selected alleles, and the resulting local reduction in polymorphism extends widely across chromosomes. A pervasive effect of linked selection might explain why heterozygosity never reaches extremely high values, even when *N_e_* is very large and drift negligible.

Distinguishing between the demographic and linked selection hypotheses is not an easy task since the 2 categories of models often make similar predictions (Wright and Gaut 2005; Schrider et al. 2016; Wang et al. 2020; Johri et al. 2022). Understanding the variation in genetic diversity, both within and among genomes, remains an important, central goal of the field of molecular evolution, and the neutralist/selectionist debate underlies this vivid body of literature. The issue, however, is no longer phrased in terms of corroborating versus challenging the neutral theory, but rather in terms of quantifying the relative contribution of neutral and selective processes to the observed patterns.

## Substitution Rates and Protein Adaptation

The second, major argument at the basis of the (nearly) neutral theory relies on the approximate clock-like evolution of protein sequences. This would appear unexpected if adaptation was governing protein evolution—phenotypes do not evolve at a constant rate. A constant rate, however, appears consistent with a prediction of the neutral model, which is that the substitution rate is independent of *N_e_* and equal to the mutation rate—which can plausibly be assumed to be approximately constant. This result, associated with the observations that synonymous substitutions are more common than nonsynonymous ones and that the amino acid composition of proteins reflects the nucleotide composition of genomes, led to the suggestion that most amino acid substitutions might be neutral or weakly selected and driven by genetic drift (Kimura 1969; King and Jukes 1969; Ohta 1973).

The rate of protein evolution and its meaning in terms of neutral versus selected evolution have been heavily debated over the last 50 yr. Gillespie (1989) repeatedly argued that the protein evolutionary rate is overdispersed—i.e. not perfectly clock-like—and that just a modest departure from the molecular clock hypothesis is expected under a number of models in which selection is the main driver. His 1989 article in *Molecular Biology and Evolution* was one of the last to explicitly focus on the validity of the rationale that initially underpinned the neutral theory. The debate took another dimension after the suggestion was made to approach the rate of adaptive amino acid substitution by comparing polymorphism and divergence patterns (McDonald and Kreitmann 1991). Elaborate statistical methods exploiting this idea were developed (Eyre-Walker and Keightley 2009; Messer and Petrov 2013) and applied to various species of microbes, plants, and animals. This led to estimates of the fraction of adaptive amino acid substitution that varied from nearly zero (Gossmann et al. 2010) up to roughly 90% (Galtier 2016)—an impressively wide range, likely in part explained by differences in the number and nature of analyzed genes, as well as in methodological choices.

Despite these uncertainties, it is a fact that, in many taxa, the ratio of nonsynonymous to synonymous changes is higher between species than within species, despite the probable existence of slightly deleterious mutations, whose fixation probability is lower than that for neutral mutations. This strongly suggests that a substantial fraction of amino acid substitutions are nonneutral, contradicting early postulates by the founders of the neutral theory. This, in turn, opens a new issue, which is the interpretation of the excess of nonsynonymous substitutions, compared with the nearly neutral expectation. A fraction of these substitutions probably reflects the response of the proteome to environmental changes, as is usually considered. Some may instead correspond to compensatory substitutions, i.e. changes restoring the function of a protein previously impaired by a population bottleneck, hitchhiking, or selfish processes such as GC-biased gene conversion (gBGC; Bolivar et al. 2018; Latrille et al. 2023). Quantifying the contribution of these many processes to the overall (or apparent) adaptive rate is an exciting challenge for the forthcoming years. Of note, the theory predicts that, if a mutation does limit adaptation, beneficial mutations should accumulate at a faster rate in large than in small *N_e_* species—a prediction that received some empirical support (Gossmann et al. 2012; Rousselle et al. 2020).

Another interesting aspect is the determination of the among-lineage variation in substitution rate. The nearly neutral theory predicts that the rate of amino acid substitution should be negatively correlated to *N_e_*—in small populations, drift is expected to reduce the efficacy of selection, thus allowing more mildly deleterious mutations to reach fixation (Ohta 1973; Lanfear et al. 2014). *N_e_* in natural populations is hard to measure but is likely correlated to traits such as body size, longevity, or reproductive systems. Empirical analyses in plants and animals have provided extensive evidence that the ratio of nonsynonymous to synonymous changes is indeed higher in presumably small *N_e_* than in presumably large *N_e_* species, which is in agreement with the nearly neutral theory (Bromham and Leys 2005; Nabholz et al. 2013; Figuet et al. 2016; Chen et al. 2017). Therefore, we have reached this peculiar situation where the analysis of amino acid substitution rates has both led to a clear rejection of a major claim of the neutral theory—that most observable changes are neutral—and at the same time indisputably demonstrated the importance of drift in protein evolution.

Of note, this research was sustained by a remarkable effort to develop models of sequence evolution and statistical methods of substitution rate estimation (e.g. Thorne et al. 1998; Yang and Nielsen 2008; Lartillot and Poujol 2011), which, in turn, opened exciting opportunities such as, among other things, the characterization of the distribution of fitness effects of mutations (Castellano et al. 2019), the reconstruction of ancestral *N_e_* and ancestral traits (Brevet and Lartillot 2021), and the connection between the biochemistry and the evolution of amino acids and proteins (Glaser et al. 2003; Goldstein 2013).

## Genome Size and the Drift Barrier Hypothesis

The neutralist/selectionist debate has not been restricted to the topics that gave rise to the neutral theory. The exploration of the growing amount of genomic data led to a number of discoveries that fueled the controversy. As noncoding DNA sequences became available, it appeared that the effects of *N_e_* on genome evolution are not limited to proteins. Regulatory sequences, such as promoters, are evolutionarily conserved among distant species in relatively large *N_e_* species such as rodents but decay rapidly in small *N_e_* species like primates (Keightley et al. 2005). Besides sequences, the way the genome functions also seems to respond to variation in *N_e_*. The drift barrier hypothesis generally states that the efficiency of the machinery regulating genome biology—e.g. DNA replication, repair, transcription, and splicing—is determined by *N_e_*, i.e. optimal in large *N_e_* species and suboptimal in small *N_e_* ones (Lynch 2011; Sung et al. 2012). Analyses of patterns of gene expression (Brawand et al. 2011; Meer et al. 2020) and alternative splicing (Bénitière et al. 2023) tend to be consistent with this advanced version of the nearly neutral theory. Codon usage bias is another good illustration: early analyses in large *N_e_* bacteria and yeasts uncovered the existence of selection for synonymous codon choice in highly expressed genes, implying a finely tuned translation machinery, whereas codon usage in large vertebrates was found to be mainly determined by the genomic context and independent of gene function (Sharp et al. 1995).

Lynch (2006, 2007, 2012) argued that the complex architecture of eukaryotic genomes and proteomes—a large size, the presence of introns, repeated elements, and numerous multimeric protein complexes—results from their reduced *N_e_*, compared with prokaryotes. This claim, however, was criticized based on the observation that *N_e_* is not a good predictor of genome size in prokaryotes (Batut et al. 2014) and that phylogenetic nonindependence is not being properly accounted for (Whitney et al. 2011). In animals, genome size has been found to respond to *N_e_* in some (Lefébure et al. 2017; Fuselli et al. 2023) but not all (Mohlhenrich and Mueller 2016; Roddy et al. 2021) of the examined contrasts, and analyses in plants yielded similarly equivocal results (Whitney et al. 2010; Bromham et al. 2015). It is puzzling to note that, >50 yr after the so-called C-paradox was coined, we still have no good understanding of the forces governing the evolution of genome size (Elliott and Gregory 2015). One difficulty could be that the types of mutations affecting genome size are diverse, apply at various time scales, and might be pleiotropic. The appearance of a novel, active transposable element, a whole-genome duplication, or a mutation modifying the efficiency of structural change repair, for instance, should influence genome size in the long run but will likely be selected on other grounds. Such mutations might be under strong selection, and their evolution, therefore, is largely independent of *N_e_*. Small insertions or deletions of noncoding DNA, on the other hand, presumably have extremely small fitness consequences and might behave effectively neutrally across a wide range of the existing *N_e_*s. This might explain why the drift barrier hypothesis fails to generally predict the among-species variation in genome size. Experimental assessments of the fitness consequences of a change in genome size (e.g. Stelzer et al. 2023) might help progress here.

## Base Composition and gBGC

GC content varies considerably within and among genomes, an observation that has intrigued molecular evolutionary scientists for decades. Interest in this topic has probably been heightened by the fact that the human genome shows a particularly high variance in GC content, which varies from ∼30% to ∼60% at a 100-kb scale. The causes of this pattern, at that time called the isochore structure, was the subject of a vigorous debate in the 1980s and the 1990s. The discovery that, among vertebrates, the warm-blooded mammals and birds, but not the cold-blooded fish and amphibians, harbored isochores led to the suggestion that this trait was an adaptation to homeothermy (Bernardi and Bernardi 1986). This hypothesis lasted for a remarkably long time, despite receiving no empirical support (Hughes et al. 1999; Belle et al. 2002) and being theoretically implausible (Piganeau et al. 2001). The climax was reached when the first DNA sequence polymorphism data in mammals revealed a segregation bias: GC alleles appeared to be more frequent, on average, than AT alleles (Eyre-Walker 1999), and especially so in the GC-richest regions of the human genome (Duret et al. 2002), suggesting that isochores were selected—a confusing situation.

The solution came from a hitherto little-documented molecular process: gBGC. gBGC is a recombination-associated transmission distorter, by which AT versus GC heterozygotes produce more GC-bearing than AT-bearing gametes, conferring a population advantage to GC alleles (Eyre-Walker 1993; Galtier et al. 2001). The last 2 decades have demonstrated that gBGC is pervasive and the major determinant of GC content variation in all domains of life (e.g. Pessia et al. 2012; Lassalle et al. 2015; Clément et al. 2017; Galtier et al. 2018; Boman et al. 2021). In vertebrates, the across-species variation in mean and variance of genomic GC content (“isochores”) was found to be mainly determined by the distribution and dynamics of the recombination rate, which, in turn, is influenced by karyotype evolution, small chromosomes experiencing a higher per base pair recombination rate (Mugal et al. 2015).

gBGC is neutral in the sense that it is unrelated to the fitness of organisms. gBGC, however, leaves a population genetic signature that resembles that of selection, and for this reason, it is a major confounding factor to be accounted for (Ratnakumar et al. 2010). The expected strength of gBGC depends on the product of the transmission bias by *N_e_*—just like selection, gBGC is less effective if drift is strong. A phylogenetic analysis of GC content variation can, therefore, inform ancient recombination maps and ancient *N_e_* (Lartillot 2013). The origin and evolution of gBGC is an open issue. Why did this meiotic bias evolve in the first place? How does its strength remain moderate across taxa differing in *N_e_* by orders of magnitude? These are promising avenues of research requiring an explicit incorporation of gBGC in evolutionary genomic models (Capra et al. 2013; Borges et al. 2019). gBGC is an important evolutionary force that had gone unnoticed for decades and was revealed thanks to the failure of the preexisting hypotheses, both neutral and nonneutral—perhaps one of the greatest successes of the neutralist/selectionist controversy.

## Why a Controversy?

In the late 1960s, despite the tiny amount of data at their disposal, a few brilliant geneticists correctly realized that the classical view of evolution, in which natural selection is the dominant force, does not apply as is at a molecular level and that the role of neutral mutations and drift had been underestimated. This marked a critical turning point in molecular evolutionary thinking. Enthused by their discovery, they slightly overstated the prevalence of neutrality. With the gradual accumulation of DNA sequence data, subsequent research converged toward a more balanced view of molecular evolution, the relative contribution of neutral and nonneutral forces being assessed with increasing accuracy (Pouyet and Gilbert 2021) for an increasingly wide range of biological problems (Ohta 2011; Austerlitz and Heyer 2018; Cannataro and Townsend 2018; Yoder et al. 2018).

The above looks like a classical process of collective knowledge advancement, of which the early stages could have been largely forgotten. Instead, the neutral and nearly neutral theories have polarized the field for half a century (Ohta and Gillespie 1996) and still arouse strong feelings (Graur et al. 2013; Kern and Hahn 2018; Jensen et al. 2019). Why is this so? The limited data sets available for decades probably played a role—it is easy to take extreme or dogmatic positions when no data can contradict them. It could also be that scientists working on relatively small *N_e_* species, such as large vertebrates, tend to adhere to the neutral theory, whereas, say, drosophilists or microbiologists might be more attracted to hypotheses involving selection—simply because drift has a limited impact in these taxa. Below, I consider yet another hypothesis and speculate that the duration and heat of the controversy might in part be explained by cultural differences among scientists.

There are several pathways for a researcher to get to study evolution at the molecular level. Many are passionate about nature and biodiversity and use DNA as a tool to better understand how organisms function and adapt to their environment. For them, natural selection is what we want to study, and building a neutralist theory is just pointless. These researchers may be frustrated by the feedback from picky reviewers who repeatedly ask for more controls and show no apparent interest in their results. Others, instead, are fascinated by genomes as biological objects and aim at understanding why they are what they are. For them, ignoring neutral and nearly neutral processes is a logical flaw, or even scientific malpractice. These scientists can be irritated by colleagues easily jumping to selectionist explanations, which often attract more attention than their subsequent refutation.

This author cannot help considering that scientists from the latter category get closer to the truth, while also recognizing that the imagination and diligence of more phenotype-oriented researchers brings exciting hypotheses to the test. Controversy is not bad, after all, and the plurality of viewpoints makes molecular evolutionary biology a lively field to work in, as illustrated year after year by the content of SMBE journals and meetings.

## References

[evae003-B1] Austerlitz F, Heyer E. Neutral theory: from complex population history to natural selection and sociocultural phenomena in human populations. Mol Biol Evol. 2018:35(6):1304–1307. 10.1093/molbev/msy067. 29659992

[evae003-B2] Barroso GV, Dutheil JY. The landscape of nucleotide diversity in *Drosophila melanogaster* is shaped by mutation rate variation. Peer Commun J. 2023:3:e40. 10.24072/pcjournal.267.

[evae003-B3] Batut B, Knibbe C, Marais G, Daubin V. Reductive genome evolution at both ends of the bacterial population size spectrum. Nat Rev Microbiol. 2014:12(12):841–850. 10.1038/nrmicro3331. 25220308

[evae003-B4] Begun DJ, Aquadro CF. Levels of naturally occurring DNA polymorphism correlate with recombination rates in *D. melanogaster*. Nature. 1992:356(6369):519–520. 10.1038/356519a0. 1560824

[evae003-B5] Belle EM, Smith N, Eyre-Walker A. Analysis of the phylogenetic distribution of isochores in vertebrates and a test of the thermal stability hypothesis. J Mol Evol. 2002:55(3):356–363. 10.1007/s00239-002-2333-1. 12187388

[evae003-B6] Benitiere F, Necsulea A, Duret A. Random genetic drift sets an upper limit on mRNA splicing accuracy in metazoans. bioRxiv 519597. 10.1101/2022.12.09.519597, 26 September 2023, preprint: not peer reviewed. PMC10932544 38470242

[evae003-B7] Bernardi G, Bernardi G. Compositional constraints and genome evolution. J Mol Evol. 1986:24(1-2):1–11. 10.1007/BF02099946. 3104608

[evae003-B8] Bolívar P, Mugal CF, Rossi M, Nater A, Wang M, Dutoit L, Ellegren H. Biased inference of selection due to GC-biased gene conversion and the rate of protein evolution in flycatchers when accounting for it. Mol Biol Evol. 2018:35(10):2475–2486. 10.1093/molbev/msy149. 30085180 PMC6188562

[evae003-B9] Boman J, Mugal CF, Backström N. The effects of GC-biased gene conversion on patterns of genetic diversity among and across butterfly genomes. Genome Biol Evol. 2021:13(5):evab064. 10.1093/gbe/evab064. 33760095 PMC8175052

[evae003-B10] Borges R, Szöllősi GJ, Kosiol C. Quantifying GC-biased gene conversion in great ape genomes using polymorphism-aware models. Genetics. 2019:212(4):1321–1336. 10.1534/genetics.119.302074. 31147380 PMC6707462

[evae003-B11] Brawand D, Soumillon M, Necsulea A, Julien P, Csárdi G, Harrigan P, Weier M, Liechti A, Aximu-Petri A, Kircher M, et al The evolution of gene expression levels in mammalian organs. Nature. 2011:478(7369):343–348. 10.1038/nature10532. 22012392

[evae003-B12] Brevet M, Lartillot N. Reconstructing the history of variation in effective population size along phylogenies. Genome Biol Evol. 2021:13(8):evab150. 10.1093/gbe/evab150. 34190972 PMC8358220

[evae003-B13] Bromham L, Hua X, Lanfear R, Cowman PF. Exploring the relationships between mutation rates, life history, genome size, environment, and species richness in flowering plants. Am Nat. 2015:185(4):507–524. 10.1086/680052. 25811085

[evae003-B14] Bromham L, Leys R. Sociality and the rate of molecular evolution. Mol Biol Evol. 2005:22(6):1393–1402. 10.1093/molbev/msi133. 15758201

[evae003-B15] Buffalo V . Quantifying the relationship between genetic diversity and population size suggests natural selection cannot explain Lewontin's paradox. Elife. 2021:10:e67509. 10.7554/eLife.67509. 34409937 PMC8486380

[evae003-B16] Cannataro VL, Townsend JP. Neutral theory and the somatic evolution of cancer. Mol Biol Evol. 2018:35(6):1308–1315. 10.1093/molbev/msy079. 29684198 PMC5967571

[evae003-B17] Capra JA, Hubisz MJ, Kostka D, Pollard KS, Siepel A. A model-based analysis of GC-biased gene conversion in the human and chimpanzee genomes. PLoS Genet. 2013:9(8):e1003684. 10.1371/journal.pgen.1003684. 23966869 PMC3744432

[evae003-B18] Castellano D, James J, Eyre-Walker A. Nearly neutral evolution across the *Drosophila melanogaster* genome. Mol Biol Evol. 2018:35(11):2685–2694. 10.1093/molbev/msy164. 30418639

[evae003-B19] Castellano D, Macià MC, Tataru P, Bataillon T, Munch K. Comparison of the full distribution of fitness effects of new amino acid mutations across great apes. Genetics. 2019:213(3):953–966. 10.1534/genetics.119.302494. 31488516 PMC6827385

[evae003-B20] Charlesworth B, Jensen JD. How can we resolve Lewontin's paradox? Genome Biol Evol. 2022:14(7):evac096. 10.1093/gbe/evac096. 35738021 PMC9305300

[evae003-B21] Charlesworth B, Morgan MT, Charlesworth D. The effect of deleterious mutations on neutral molecular variation. Genetics. 1993:134(4):1289–1303. 10.1093/genetics/134.4.1289. 8375663 PMC1205596

[evae003-B22] Chen J, Glémin S, Lascoux M. Genetic diversity and the efficacy of purifying selection across plant and animal species. Mol Biol Evol. 2017:34(6):1417–1428. 10.1093/molbev/msx088. 28333215

[evae003-B23] Chen J, Glémin S, Lascoux M. From drift to draft: how much do beneficial mutations actually contribute to predictions of Ohta's slightly deleterious model of molecular evolution? Genetics. 2020:214(4):1005–1018. 10.1534/genetics.119.302869. 32015019 PMC7153929

[evae003-B24] Clément Y, Sarah G, Holtz Y, Homa F, Pointet S, Contreras S, Nabholz B, Sabot F, Sauné L, Ardisson M, et al Evolutionary forces affecting synonymous variations in plant genomes. PLoS Genet. 2017:13(5):e1006799. 10.1371/journal.pgen.1006799. 28531201 PMC5460877

[evae003-B25] Corbett-Detig RB, Hartl DL, Sackton TB. Natural selection constrains neutral diversity across a wide range of species. PLoS Biol. 2015:13(4):e1002112. 10.1371/journal.pbio.1002112. 25859758 PMC4393120

[evae003-B26] Duret L, Semon M, Piganeau G, Mouchiroud D, Galtier N. Vanishing GC-rich isochores in mammalian genomes. Genetics. 2002:162(4):1837–1847. 10.1093/genetics/162.4.1837. 12524353 PMC1462357

[evae003-B27] Elliott TA, Gregory TR. What's in a genome? The C-value enigma and the evolution of eukaryotic genome content. Philos Trans R Soc Lond B Biol Sci. 2015:370(1678):20140331. 10.1098/rstb.2014.0331. 26323762 PMC4571570

[evae003-B28] Elyashiv E, Sattath S, Hu TT, Strutsovsky A, McVicker G, Andolfatto P, Coop G, Sella G. A genomic map of the effects of linked selection in Drosophila. PLoS Genet. 2016:12(8):e1006130. 10.1371/journal.pgen.1006130. 27536991 PMC4990265

[evae003-B29] Enard D, Messer PW, Petrov DA. Genome-wide signals of positive selection in human evolution. Genome Res. 2014:24(6):885–895. 10.1101/gr.164822.113. 24619126 PMC4032853

[evae003-B30] Eyre-Walker A . Recombination and mammalian genome evolution. Proc Biol Sci. 1993:252(1335):237–243. 10.1098/rspb.1993.0071. 8394585

[evae003-B31] Eyre-Walker A . Evidence of selection on silent site base composition in mammals: potential implications for the evolution of isochores and junk DNA. Genetics. 1999:152(2):675–683. 10.1093/genetics/152.2.675. 10353909 PMC1460637

[evae003-B32] Eyre-Walker A, Keightley PD. Estimating the rate of adaptive molecular evolution in the presence of slightly deleterious mutations and population size change. Mol Biol Evol. 2009:26(9):2097–2108. 10.1093/molbev/msp119. 19535738

[evae003-B33] Figuet E, Nabholz B, Bonneau M, Mas Carrio E, Nadachowska-Brzyska K, Ellegren H, Galtier N. Life history traits, protein evolution, and the nearly neutral theory in amniotes. Mol Biol Evol. 2016:33(6):1517–1527. 10.1093/molbev/msw033. 26944704

[evae003-B34] Filatov DA . Extreme Lewontin's paradox in ubiquitous marine phytoplankton species. Mol Biol Evol. 2019:36(1):4–14. 10.1093/molbev/msy195. 30351418

[evae003-B35] Fuselli S, Greco S, Biello R, Palmitessa S, Lago M, Meneghetti C, McDougall C, Trucchi E, Rota Stabelli O, Biscotti AM, et al Relaxation of natural selection in the evolution of the giant lungfish genomes. Mol Biol Evol. 2023:40(9):msad193. 10.1093/molbev/msad193. 37671664 PMC10503785

[evae003-B36] Galtier N . Adaptive protein evolution in animals and the effective population size hypothesis. PLoS Genet. 2016:12(1):e1005774. 10.1371/journal.pgen.1005774. 26752180 PMC4709115

[evae003-B37] Galtier N, Piganeau G, Mouchiroud D, Duret L. GC-content evolution in mammalian genomes: the biased gene conversion hypothesis. Genetics. 2001:159(2):907–911. 10.1093/genetics/159.2.907. 11693127 PMC1461818

[evae003-B38] Galtier N, Roux C, Rousselle M, Romiguier J, Figuet E, Glémin S, Bierne N, Duret L. Codon usage bias in animals: disentangling the effects of natural selection, effective population size, and GC-biased gene conversion. Mol Biol Evol. 2018:35(5):1092–1103. 10.1093/molbev/msy015. 29390090

[evae003-B39] Gillespie JH . Lineage effects and the index of dispersion of molecular evolution. Mol Biol Evol. 1989:6(6):636–647. 10.1093/oxfordjournals.molbev.a040576. 2488476

[evae003-B40] Glaser F, Pupko T, Paz I, Bell RE, Bechor-Shental D, Martz E, Ben-Tal N. ConSurf: identification of functional regions in proteins by surface-mapping of phylogenetic information. Bioinformatics. 2003:19(1):163–164. 10.1093/bioinformatics/19.1.163. 12499312

[evae003-B41] Goldstein RA . Population size dependence of fitness effect distribution and substitution rate probed by biophysical model of protein thermostability. Genome Biol Evol. 2013:5(9):1584–1593. 10.1093/gbe/evt110. 23884461 PMC3787666

[evae003-B42] Gossmann TI, Keightley PD, Eyre-Walker A. The effect of variation in the effective population size on the rate of adaptive molecular evolution in eukaryotes. Genome Biol Evol. 2012:4(5):658–667. 10.1093/gbe/evs027. 22436998 PMC3381672

[evae003-B43] Gossmann TI, Song BH, Windsor AJ, Mitchell-Olds T, Dixon CJ, Kapralov MV, Filatov DA, Eyre-Walker A. Genome wide analyses reveal little evidence for adaptive evolution in many plant species. Mol Biol Evol. 2010:27(8):1822–1832. 10.1093/molbev/msq079. 20299543 PMC2915642

[evae003-B44] Graur D, Zheng Y, Price N, Azevedo RB, Zufall RA, Elhaik E. On the immortality of television sets: “function” in the human genome according to the evolution-free gospel of ENCODE. Genome Biol Evol. 2013:5(3):578–590. 10.1093/gbe/evt028. 23431001 PMC3622293

[evae003-B45] Hughes S, Zelus D, Mouchiroud D. Warm-blooded isochore structure in Nile crocodile and turtle. Mol Biol Evol. 1999:16(11):1521–1527. 10.1093/oxfordjournals.molbev.a026064. 10555283

[evae003-B46] Jensen JD, Payseur BA, Stephan W, Aquadro CF, Lynch M, Charlesworth D, Charlesworth B. The importance of the neutral theory in 1968 and 50 years on: a response to Kern and Hahn 2018. Evolution. 2019:73(1):111–114. 10.1111/evo.13650. 30460993 PMC6496948

[evae003-B47] Johri P, Eyre-Walker A, Gutenkunst RN, Lohmueller KE, Jensen JD. On the prospect of achieving accurate joint estimation of selection with population history. Genome Biol Evol. 2022:14(7):evac088. 10.1093/gbe/evac088. 35675379 PMC9254643

[evae003-B48] Keightley PD, Lercher MJ, Eyre-Walker A. Evidence for widespread degradation of gene control regions in hominid genomes. PLoS Biol. 2005:3(2):e42. 10.1371/journal.pbio.0030042. 15678168 PMC544929

[evae003-B49] Kern AD, Hahn MW. The neutral theory in light of natural selection. Mol Biol Evol. 2018:35(6):1366–1371. 10.1093/molbev/msy092. 29722831 PMC5967545

[evae003-B50] Kimura M . Evolutionary rate at the molecular level. Nature. 1968:217(5129):624–626. 10.1038/217624a0. 5637732

[evae003-B51] Kimura M . The rate of molecular evolution considered from the standpoint of population genetics. Proc Natl Acad Sci U S A. 1969:63(4):1181–1188. 10.1073/pnas.63.4.1181. 5260917 PMC223447

[evae003-B52] Kimura M . Theoretical foundation of population genetics at the molecular level. Theor Popul Biol. 1971:2(2):174–208. 10.1016/0040-5809(71)90014-1. 5162686

[evae003-B53] King JL, Jukes TH. Non-Darwinian evolution. Science. 1969:164(3881):788–798. 10.1126/science.164.3881.788. 5767777

[evae003-B54] Lanfear R, Kokko H, Eyre-Walker A. Population size and the rate of evolution. Trends Ecol Evol. 2014:29(1):33–41. 10.1016/j.tree.2013.09.009. 24148292

[evae003-B55] Lartillot N . Phylogenetic patterns of GC-biased gene conversion in placental mammals and the evolutionary dynamics of recombination landscapes. Mol Biol Evol. 2013:30(3):489–502. 10.1093/molbev/mss239. 23079417

[evae003-B56] Lartillot N, Poujol R. A phylogenetic model for investigating correlated evolution of substitution rates and continuous phenotypic characters. Mol Biol Evol. 2011:28(1):729–744. 10.1093/molbev/msq244. 20926596

[evae003-B57] Lassalle F, Périan S, Bataillon T, Nesme X, Duret L, Daubin V. GC-content evolution in bacterial genomes: the biased gene conversion hypothesis expands. PLoS Genet. 2015:11(2):e1004941. 10.1371/journal.pgen.1004941. 25659072 PMC4450053

[evae003-B58] Latrille T, Joseph J, Hartasánchez DA, Salamin N. Mammalian protein-coding genes exhibit widespread beneficial mutations that are not adaptive. bioRxiv 538864. 10.1101/2023.05.03.538864, 4 May 2023, preprint: not peer reviewed

[evae003-B59] Lefébure T, Morvan C, Malard F, François C, Konecny-Dupré L, Guéguen L, Weiss-Gayet M, Seguin-Orlando A, Ermini L, Sarkissian C, et al Less effective selection leads to larger genomes. Genome Res. 2017:27(6):1016–1028. 10.1101/gr.212589.116. 28424354 PMC5453316

[evae003-B60] Leffler EM, Bullaughey K, Matute DR, Meyer WK, Ségurel L, Venkat A, Andolfatto P, Przeworski M. Revisiting an old riddle: what determines genetic diversity levels within species? PLoS Biol. 2012:10(9):e1001388. 10.1371/journal.pbio.1001388. 22984349 PMC3439417

[evae003-B61] Lewontin RC . The genetic basis of evolutionary change. New York (NY): Columbia University Press; 1974.

[evae003-B62] Lynch M . The origins of eukaryotic gene structure. Mol Biol Evol. 2006:23(2):450–468. 10.1093/molbev/msj050. 16280547

[evae003-B63] Lynch M . The origins of genome architecture. Sunderland: Sinauer; 2007.

[evae003-B64] Lynch M . The lower bound to the evolution of mutation rates. Genome Biol Evol. 2011:3:1107–1118. 10.1093/gbe/evr066. 21821597 PMC3194889

[evae003-B65] Lynch M . The evolution of multimeric protein assemblages. Mol Biol Evol. 2012:29(5):1353–1366. 10.1093/molbev/msr300. 22144639 PMC3339316

[evae003-B66] Maynard Smith J, Haigh J. The hitch-hiking effect of a favourable gene. Genet Res. 1974:23(1):23–35. 10.1017/S0016672300014634. 4407212

[evae003-B67] McDonald JH, Kreitman M. Adaptive protein evolution at the *Adh* locus in *Drosophila*. Nature. 1991:351(6328):652–654. 10.1038/351652a0. 1904993

[evae003-B68] Meer KM, Nelson PG, Xiong K, Masel J. High transcriptional error rates vary as a function of gene expression level. Genome Biol Evol. 2020:12(1):3754–3761. 10.1093/gbe/evz275. 31841128 PMC6988749

[evae003-B69] Messer PW, Petrov DA. Frequent adaptation and the McDonald-Kreitman test. Proc Natl Acad Sci U S A. 2013:110(21):8615–8620. 10.1073/pnas.1220835110. 23650353 PMC3666677

[evae003-B70] Mohlhenrich ER, Mueller RL. Genetic drift and mutational hazard in the evolution of salamander genomic gigantism. Evolution. 2016:70(12):2865–2878. 10.1111/evo.13084. 27714793

[evae003-B71] Mugal CF, Weber CC, Ellegren H. GC-biased gene conversion links the recombination landscape and demography to genomic base composition: GC-biased gene conversion drives genomic base composition across a wide range of species. Bioessays. 2015:37(12):1317–1326. 10.1002/bies.201500058. 26445215

[evae003-B72] Nabholz B, Uwimana N, Lartillot N. Reconstructing the phylogenetic history of long-term effective population size and life-history traits using patterns of amino acid replacement in mitochondrial genomes of mammals and birds. Genome Biol Evol. 2013:5(7):1273–1290. 10.1093/gbe/evt083. 23711670 PMC3730341

[evae003-B73] Nachman MW . Single nucleotide polymorphisms and recombination rate in humans. Trends Genet. 2001:17(9):481–485. 10.1016/S0168-9525(01)02409-X. 11525814

[evae003-B74] Ohta T . Associative overdominance caused by linked detrimental mutations. Genet Res. 1971:18(3):277–286. 10.1017/S0016672300012684. 5158298

[evae003-B75] Ohta T . Slightly deleterious mutant substitutions in evolution. Nature. 1973:246(5428):96–98. 10.1038/246096a0. 4585855

[evae003-B76] Ohta T . Near-neutrality, robustness, and epigenetics. Genome Biol Evol. 2011:3:1034–1038. 10.1093/gbe/evr012. 21979156 PMC3227401

[evae003-B77] Ohta T, Gillespie JH. Development of neutral and nearly neutral theories. Theor Popul Biol. 1996:49(2):128–142. 10.1006/tpbi.1996.0007. 8813019

[evae003-B78] Pessia E, Popa A, Mousset S, Rezvoy C, Duret L, Marais GA. Evidence for widespread GC-biased gene conversion in eukaryotes. Genome Biol Evol. 2012:4(7):675–682. 10.1093/gbe/evs052. 22628461 PMC5635611

[evae003-B79] Piganeau G, Westrelin R, Tourancheau B, Gautier C. Multiplicative versus additive selection in relation to genome evolution: a simulation study. Genet Res. 2001:78(2):171–175. 10.1017/S0016672301005249. 11732094

[evae003-B80] Pouyet F, Gilbert KJ. Towards an improved understanding of molecular evolution: the relative roles of selection, drift, and everything in between. Peer Commun J. 2021:1:e27. 10.24072/pcjournal.16.

[evae003-B81] Ratnakumar A, Mousset S, Glémin S, Berglund J, Galtier N, Duret L, Webster MT. Detecting positive selection within genomes: the problem of biased gene conversion. Philos Trans R Soc Lond B Biol Sci. 2010:365(1552):2571–2580. 10.1098/rstb.2010.0007. 20643747 PMC2935097

[evae003-B82] Roddy AB, Alvarez-Ponce D, Roy SW. Mammals with small populations do not exhibit larger genomes. Mol Biol Evol. 2021:38(9):3737–3741. 10.1093/molbev/msab142. 33956142 PMC8382904

[evae003-B83] Romiguier J, Gayral P, Ballenghien M, Bernard A, Cahais V, Chenuil A, Chiari Y, Dernat R, Duret L, Faivre N, et al Comparative population genomics in animals uncovers the determinants of genetic diversity. Nature. 2014:515(7526):261–263. 10.1038/nature13685. 25141177

[evae003-B84] Rousselle M, Simion P, Tilak MK, Figuet E, Nabholz B, Galtier N. Is adaptation limited by mutation? A timescale-dependent effect of genetic diversity on the adaptive substitution rate in animals. PLoS Genet. 2020:16(4):e1008668. 10.1371/journal.pgen.1008668. 32251427 PMC7162527

[evae003-B99] Russo CAM, Eyre-Walker A, Katz LA, Gaut BS. Forty years of inferential methods in the Journals of the Society for Molecular Biology and Evolution. Mol Biol Evol. 2024:41(1):msad264. 10.1093/molbev/msad264. 38197288 PMC10763999

[evae003-B85] Schrider DR, Shanku AG, Kern AD. Effects of linked selective sweeps on demographic inference and model selection. Genetics. 2016:204(3):1207–1223. 10.1534/genetics.116.190223. 27605051 PMC5105852

[evae003-B86] Sharp PM, Averof M, Lloyd AT, Matassi G, Peden JF. DNA sequence evolution: the sounds of silence. Philos Trans R Soc Lond B Biol Sci. 1995:349(1329):241–247. 10.1098/rstb.1995.0108. 8577834

[evae003-B87] Smith TCA, Arndt PF, Eyre-Walker A. Large scale variation in the rate of germ-line de novo mutation, base composition, divergence and diversity in humans. PLoS Genet. 2018:14(3):e1007254. 10.1371/journal.pgen.1007254. 29590096 PMC5891062

[evae003-B88] Stelzer CP, Pichler M, Stadler P. Genome streamlining and clonal erosion in nutrient-limited environments: a test using genome-size variable populations. Evolution. 2023:77(11):2378–2391. 10.1093/evolut/qpad144. 37724883

[evae003-B89] Sung W, Ackerman MS, Miller SF, Doak TG, Lynch M. Drift-barrier hypothesis and mutation-rate evolution. Proc Natl Acad Sci U S A. 2012:109(45):18488–18492. 10.1073/pnas.1216223109. 23077252 PMC3494944

[evae003-B90] Thorne JL, Kishino H, Painter IS. Estimating the rate of evolution of the rate of molecular evolution. Mol Biol Evol. 1998:15(12):1647–1657. 10.1093/oxfordjournals.molbev.a025892. 9866200

[evae003-B91] Wang X, Bernhardsson C, Ingvarsson PK. Demography and natural selection have shaped genetic variation in the widely distributed Conifer Norway Spruce (*Picea abies*). Genome Biol Evol. 2020:12(2):3803–3817. 10.1093/gbe/evaa005. 31958121 PMC7046165

[evae003-B92] Whitney KD, Baack EJ, Hamrick JL, Godt MJ, Barringer BC, Bennett MD, Eckert CG, Goodwillie C, Kalisz S, Leitch IJ, et al A role for nonadaptive processes in plant genome size evolution? Evolution. 2010:64(7):2097–2109. 10.1111/j.1558-5646.2010.00967.x. 20148953

[evae003-B93] Whitney KD, Boussau B, Baack EJ, Garland T Jr. Drift and genome complexity revisited. PLoS Genet. 2011:7(6):e1002092. 10.1371/journal.pgen.1002092. 21695239 PMC3111538

[evae003-B94] Wiehe TH, Stephan W. Analysis of a genetic hitchhiking model, and its application to DNA polymorphism data from *Drosophila melanogaster*. Mol Biol Evol. 1993:10(4):842–854. 10.1093/oxfordjournals.molbev.a040046. 8355603

[evae003-B95] Wright SI, Gaut BS. Molecular population genetics and the search for adaptive evolution in plants. Mol Biol Evol. 2005:22(3):506–519. 10.1093/molbev/msi035. 15525701

[evae003-B96] Yang Z, Nielsen R. Mutation-selection models of codon substitution and their use to estimate selective strengths on codon usage. Mol Biol Evol. 2008:25(3):568–579. 10.1093/molbev/msm284. 18178545

[evae003-B97] Yoder AD, Poelstra JW, Tiley GP, Williams RC. Neutral theory is the foundation of conservation genetics. Mol Biol Evol. 2018:35(6):1322–1326. 10.1093/molbev/msy076. 29669008

